# Isolation and identification of bioactive compounds from *Antrodia camphorata* against ESKAPE pathogens

**DOI:** 10.1371/journal.pone.0293361

**Published:** 2023-10-27

**Authors:** Ya-Dong Zhang, Liang-Yan Liu, Dong Wang, Xiao-Long Yuan, Yuan Zheng, Yi Wang

**Affiliations:** 1 College of Forestry, Southwest Forestry University, Kunming, China; 2 Laboratory of Forest Plant Cultivation and Utilization, The Key Laboratory of Rare and Endangered Forest Plants of State Forestry Administration, Yunnan Academy of Forestry and Grassland, Kunming, China; 3 Yunnan Key Laboratory for Fungal Diversity and Green Development, Kunming, China; 4 College of Agronomy and Biotechnology, Yunnan Agriculture University, Kunming, Yunnan, China; University of Jeddah, SAUDI ARABIA

## Abstract

Antimicrobial resistance is a major threat to human health globally. *Antrodia camphorata* was grown in a malt/yeast extract broth liquid medium for 15 days. Then, 4-L fermentation broth was harvested, yielding 7.13 g of the ethyl acetate extract. By tracing the antimicrobial activity, 12.22 mg of the antimicrobial compound was isolated. The structure of 5-methyl-benzo [1,3]-dioxole-4,7-diol (MBBD) was elucidated using NMR and MS data analyses. The antibacterial activity of MBBD was detected through the microbroth dilution method. MBBD exhibited broad-spectrum antibacterial activity. The minimum inhibitory concentration (MIC) range of MBBD for drug-resistant pathogenic bacteria was 64–256 μg/mL, with the lowest MIC observed for *Acinetobacter baumannii* (64 μg/mL), followed by *Pseudomonas aeruginosa* (MIC = 128 μg/mL). *Klebsiella pneumoniae*, *Staphylococcus aureus*, *Enterococcus faecalis*, and *Escherichia coli* were also sensitive, with an MIC of 256 μg/mL. The MIC range of MBBD against 10 foodborne pathogens was 12.5–100 μg/mL. Based on the results of this study, MBBD exhibits broad-spectrum antibacterial activity, particularly demonstrating excellent inhibitory effects against *A*. *baumannii*. MBBD will be good candidates for new antimicrobial drugs.

## Introduction

Globally, the spread of antibiotic-resistant bacteria is a substantial threat causing morbidity and mortality [1, 2]. Drug-resistant bacteria directly caused 1.27 million deaths worldwide in 2019. As many as 4.95 million deaths were caused by drug-resistant bacterial infections, making drug resistance the third leading cause of human death [3]. ESKAPE (*Enterococcus faecalis*, *Staphylococcus aureus*, *Klebsiella pneumoniae*, *Acinetobacter baumannii*, *Pseudomonas aeruginosa*, and *Escherichia coli*) are the most common opportunistic pathogens causing nosocomial infections [4]. ESKAPE-induced infections are often associated with high morbidity, mortality, and treatment costs. These pathogens are highly mutagenic, rapidly transmissible, and exhibit severe drug resistance [5, 6]. Types and mechanisms of bacterial resistance are complex and belong to four main categories: impaired drug penetration, altered drug targets, drug inactivation, and active efflux. Notably, the majority of conventional antibiotics target specific bacterial surfaces or structures that can contribute to resistance development [7, 8]. The rate of antibiotic development has not matched with the increasing rate of bacterial resistance. Thus, new antibacterial drugs need to be urgently discovered and developed for tackling the problem of bacterial resistance. Fungi produce a diverse array of secondary metabolites, such as antimicrobial peptides, polyketides, phenolics, and terpenoids. These metabolites exhibit various antibacterial activities. For instance, chaetochromin A, a compound isolated from the secondary metabolite of *Chaetomium gracile*, exhibits significant antibacterial activity against *E*. *coli* and *S*. *aureus* [9]. Other researchers obtained Bionectin D from the ethyl acetate extract of the endophytic fungus *Bionectria sp*. Y1085, which was isolated from the plant *Huperzia serrata*. Similarly, Bionectin D exhibited significant antibacterial activity against both *E*. *coli* and *S*. *aureus* [10]. The new endophytic fungus, *Curvularia* sp. T12, was isolated from the medicinal plant *Rauwolfia macrophylla*. Large-scale fermentation and subsequent extraction of the crude extract yielded two compounds, 2’-deoxyribolactone and hexylitaconic acid. These compounds exhibited significant inhibitory effects against *E*. *coli*, *P*. *agarici*, and *S*. *warneri* [11]. Additionally, previous reports have indicated that dothideomycetide A, a compound isolated from the endophytic fungus *Dothideomycete* sp. of a Thai medicinal plant *Tiliacora triandra*, exhibits antibacterial activity against methicillin-resistant *S*. *aureus* strains [12]. However, it is worth noting that the cultivation process for fungi is concise and uncomplicated, making it economically feasible. This process also demonstrates a notable level of versatility, as it can be finely tuned through the addition of precursors, elicitors, specialized enzymes, and modifiers, thereby significantly enhancing the production of bioactive compounds [13]. The utilization of fungal metabolites for the management of bacterial infections and addressing antibacterial resistance holds promising potential.

*Antrodia camphorata* is a unique, valuable, and endemic mushroom species containing various active constituents, including terpenoids, maleic and succinic acid derivatives, benzenoids, benzoquinone derivatives, lignans, and polysaccharides [14, 15]. These secondary metabolites exhibit varied biological activities, such as anticancer, antidiabetic, immunomodulatory, anti-inflammatory, antiviral, antiallergic, and antioxidant activities [16–18]. Furthermore, *A*. *camphorata* has been reported to possess antibacterial bioactive. Methyl antcinate and antcins, which are extracted from *A*. *camphorata* fruiting bodies, have displayed potent inhibitory action against *Helicobacter pylori*, the bacterium causing gastritis [19]. Both ethyl acetate and chloroform extracts of *A*. *camphorata* significantly inhibited oral bacteria such as *Streptococcus mutants* and *Porphyromonas gingivalis* [20]. However, reports on the antibacterial activity of *A*. *camphorata* extracts against drug-resistant bacteria and foodborne pathogens are lacking. Therefore, this study investigated the antimicrobial activity of *A*. *camphorata* fermentation broth and isolated, purified, and identified the antimicrobial compounds present in the broth by using an antimicrobial activity tracking method.

## Materials and methods

### Fungal culture and growth conditions

The *A*. *camphorata* strain used in this experiment was provided by the Yunnan Academy of Forestry and Grassland Science (Kunming, Yunnan, China). The fungal species were identified using ITS, β-tubulin gene fragments, and elongation factor 1α gene fragments, with the strain designated as YAFAC008 [21]. The *A*. *camphorata* strain was inoculated in PDA and cultivated in a constant temperature incubator at 26°C with 60% humidity for 12 days, followed by storage in a refrigerator at 4°C. Subsequently, 100 mL of malt/yeast extract medium was added to each of 40 triangular flasks with a volume of 250 mL, and they were autoclaved for sterilization. After cooling to room temperature, precise 0.5 cm^2^ of *A*. *camphorata* mycelia were excised from the mycelium’s edge and placed into each triangular flask under aseptic conditions. The flasks were then placed in a shaker and incubated at 28°C with agitation at 150 r/min for 15 days (S1 Fig).

### Pathogenic bacteria and antibiotics used in antimicrobial experiments

The pathogenic bacteria used in the experiment included: drug-resistant pathogenic bacteria (*A*. *baumannii*, *P*. *aeruginosa*, *K*. *pneumoniae*, *S*. *aureus*, *E*. *faecalis*, and *E*. *coli*) that were purchased from the Guangdong Bacterial Resistance Monitoring and Quality Control Centre. The foodborne pathogens included: *Bacillus cereus* (BC), *B*. *lentus* (BL), *B*. *pumilus* (BP), *B*. *subtilis* (BS), *Streptococcus agalactiae* (SA), *Shigella flexneri* (SF), *Micrococcus luteus* (ML), *Vibrio parahaemolyticus* (VB), *Staphylococcus haemolyticus* (SH), and *Salmonella paratyphi B* (SP) were purchased from Henan Industrial Microbial Strain Engineering Technology Research Centre (S2 Table). The disease-causing bacteria were inoculated in Luria–Bertani liquid medium and incubated at 37°C for 24 h. The cultured bacteria were coated with sterile saline in a 10-fold gradient dilution for colony counting. Then, each bacterial solution was diluted with saline to a bacterial concentration of 1 × 10^6^ Colony-Forming Units per Milliliter (CFU/mL) according to the colony counting results.

The antibiotics, including chloramphenicol, gentamycin, ampicillin, streptomycin, tetracycline, and kanamycin, were purchased from Kunming Shuoyang Technology Company. Each antibiotic was weighed around 50.00 mg, placed in a centrifuge tube, and dissolved by adding 1.0 mL of Dimethyl Sulfoxide (DMSO) to ensure that the concentration of each antibiotic was 50 mg/mL.

### Preparation of *A*. *camphorata* extracts

After the incubation period, *A*. *camphorata* fermentation broth was filtered to separate the mycelium, and the pH of the broth was adjusted to 1–2 (S2 Fig). Ethyl acetate was added at a 1:1 volume ratio, shaken and mixed, sonicated for 60 min, and let stand for 8 hours. The lower layer of water was removed, and the top layer of ethyl acetate was dried using a rotary evaporator and weighed.

### Antibacterial activity of *A*. *camphorata* extracts

The paper diffusion method was used to test antibacterial activity [22]. Using a sterile cotton swab, 250 μL of the bacterial suspension was applied to a 4-mm solid agar-containing plate (diameter: 90 mm). Sterile filter paper discs (diameter: 6 mm) were affixed to the bacteria-containing agar plates, and 10 μL of the *A*. *camphorata* extract (50 mg/mL dissolved with DMSO) was added dropwise. The diameter of the clear inhibition zone (mm) around the round paper slice of the drop-spiked extract was measured after 24 h of incubation at 37°C. In all experiments, a negative control (10 μL DMSO) was included; the positive control used the aforementioned six standard antibiotics (50 mg/mL).

### Isolation and identification of compounds

In the liquid fermentation culture of 4 L of *A*. *camphorata*, the ethyl acetate extract was prepared at the end of the culture period. Equal masses of the 100–200-mesh silica gel were stirred with the extract to dry. The extract weighed 10 times the weight of the 200–300-mesh silica gel, filled in a normal phase silica gel column (length: 60 cm, diameter: 6 cm), and eluted with a gradient of petroleum ether and ethyl acetate, with 1 L of each gradient. The elute was concentrated to obtain a normal-phase silica gel column separation fraction, and this fraction was assayed separately for antibacterial activity. The fraction with antibacterial activity was used as the target fraction of the separated compounds. The target components were subjected to thin-layer chromatography, and similar components were combined. The monomeric compounds were carried out on the Agilent 1260 High-Performance Liquid Chromatography (HPLC) system (Agilent Technologies, Wilmington, Germany) equipped with an Agilent four-unit pump (CA, Germany), a 7125 injector (CA, Germany) and a G1314A UV detector. The analytical column used was a Zorbax Extend-C18 column (4.6 mm × 250 mm, 5 μm, Agilent, Germany). The absorbance was measured at 254 nm. Elution was carried out at a flow rate of 200 μL/min at 30°C. The mobile phase consisted of A (water) and B (100% acetonitrile): 0–90 min, 100%–0% A, and 0%–100% B. The pure monomeric compounds were subjected to nuclear magnetic resonance (NMR) and mass spectrometry (MS) to identify their structures. ^1^HNMR and ^13^C-NMR spectra were recorded on Bruker AVANCE III 400 MHz (Bruker BioSpin GmbH, Rheinstetten, Germany) instruments, using tetramethylsilane (TMS) as an internal standard: chemical shifts (*δ*) are given in ppm, coupling constants (*J*) in Hz, and the solvent signals were used as references (CDCl_3_: δ_C_ = 77.2 ppm, residual CHCl_3_ in CDCl_3_: δ_H_ = 7.26 ppm; CD3OD: δ_C_ = 49.0 ppm, residual CH_3_OH in CD_3_OD: δ_H_ = 4.78 ppm). MS data were obtained in the ESI mode on API Qstar Pulsar instrument.

### Determination of Minimum Inhibitory Concentration (MIC) of compound MBBD

The microbroth dilution method was used to determine the MIC of the compound MBBD against the tested bacteria [23]. MBBD dissolved in DMSO was diluted with sterile saline in a twofold gradient. Then, 100 μL of MBBD solution at different dilutions (100, 50, 25, and 12.5 μg/mL) and each suspension of foodborne pathogens were pipetted into small wells of 96-well plates to determine the MIC of MBBD against the foodborne pathogens. Later, 100 μL of MBBD solution at different dilutions (1024, 512, 256, 128, 64, 32, 16, 8, 4, 2, and 1 μg/mL) and the suspension of each drug-resistant pathogenic bacteria were pipetted into the wells of a 96-well plate to determine the MIC of MBBD against the drug-resistant pathogenic bacteria. The wells spiked with 100 μL DMSO were used as the solvent control group, the wells spiked with 100 μL sterile saline were used as the blank control group, and six replicates were established for each group. The 96-well plates were placed in a shaker and incubated at 37°C and 120 r/min for 24 h. At 600 nm, the bacterial growth levels were measured using an enzyme marker. MIC was defined as the lowest dilution of MBBD with an absorbance of ≤0.1.

### Cytotoxicity assay of MBBD

The cytotoxic activity of MBBD was tested using the MTS method [23]. The cell lines included were leukemia HL-60, lung cancer A-S49, liver cancer SMMC-7721, breast cancer MCF-7, colon cancer SW480, and human normal lung epithelial cells BEAS-2B. The cells were grown at 37°C in 5% CO2 in Dulbecco’s Modified Eagle Medium supplemented with 4.5 g/L glucose, 2 mM L-glutamine, 1% penicillin-streptomycin, and 10% fetal bovine serum. The cells were seeded at a density of 1.5 × 10^4^ cells/well in 96-well culture plates overnight and then treated with MBBD at 40 μM. After 24 h of incubation, a culture medium containing MTS (1 mg/mL) was added to each well, and the plate was incubated for another 2 h. Consecutively, the medium was removed, and DMSO was added to extract the MTS formazan. The absorbance of each well was measured using an enzyme-linked immunosorbent assay reader at 492 nm. Three blank replicate wells (a mixture of 20 μL of MTS solution and 100 μL of culture solution) and two positive controls of cisplatin DDP and taxol were used for each experiment.

### Statistical analysis

The means were compared using the SPSS for Windows statistical software package. The experiment was repeated three times, with the results presented as the mean ± standard deviation of three independent experiments.

## Results

### Comparing the antimicrobial activity of *A*. *camphorata* extracts with the positive control

**Fig 1:**
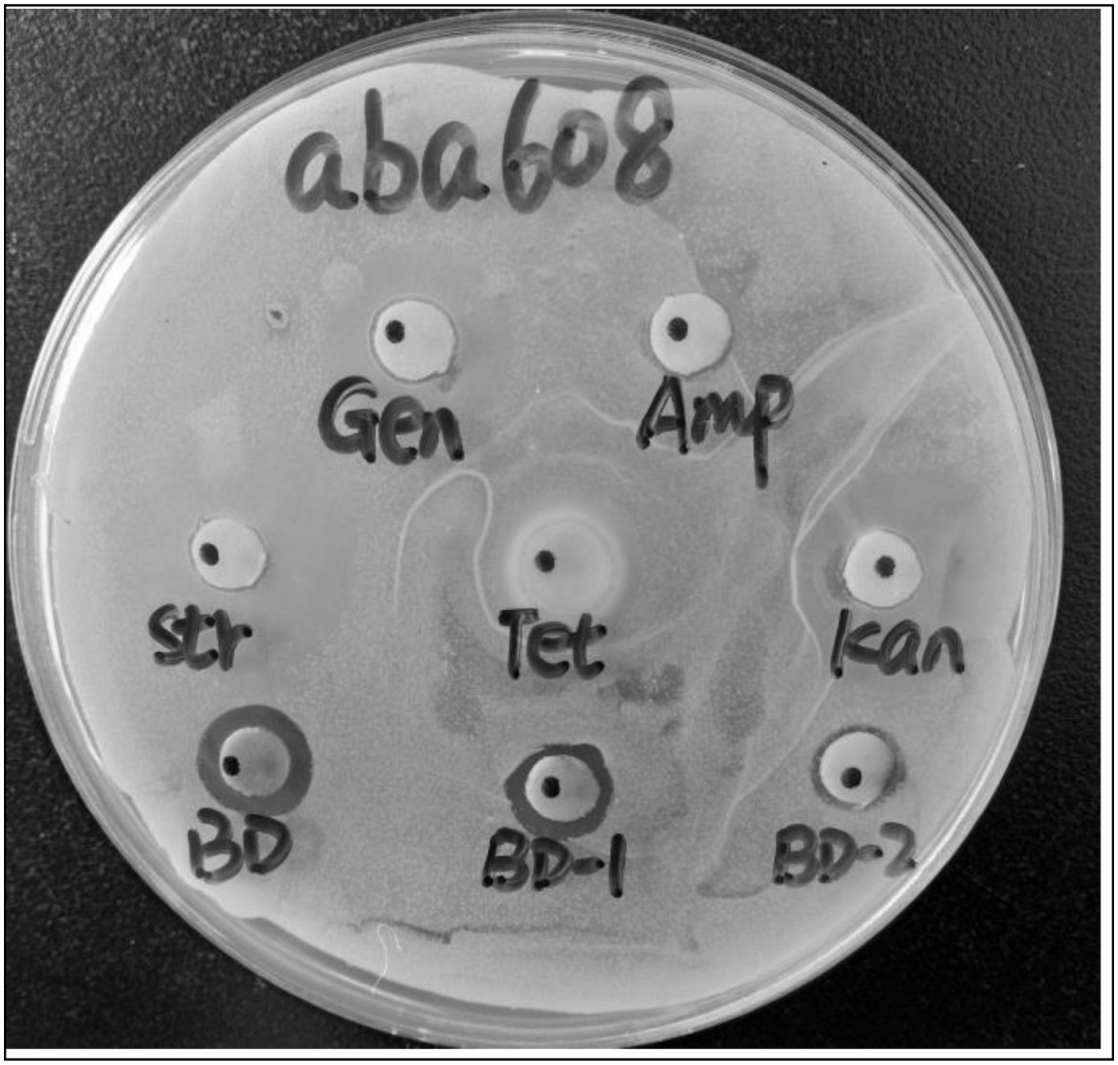
Antibacterial activity of *A*. *camphorata* extracts compared with 5 antibiotics. Gen. represents Gentamicin, Amp. represents Ampicillin, Str. represents Streptomycin, Tet. represents Tetracycline, Kan. represents Kanamycin, BD represents *A*. *camphorata* extracts (50.00 mg/mL), BD-1 represents *A*. *camphorata* extracts (25.00 mg/mL), BD-2 represents *A*. *camphorata* extracts (12.50 mg/mL).

Drug-resistant pathogenic bacteria exhibited resistance to at least one of the six tested antibiotics. All the tested bacteria demonstrated resistance to ampicillin without exhibiting any discernible inhibition zone. Multidrug resistance was defined as a lack of susceptibility to at least one agent in three or more categories of antibiotics. *A*. *baumannii* and *P*. *aeruginosa* exhibited resistance to multiple antibiotics, with resistance rates of 6/6 and 5/6, respectively. In contrast, *E*. *coli* demonstrated resistance to 2/6 antibiotics, while *S*. *aureus*, *E*. *faecalis*, and *K*. *pneumoniae* each displayed a resistance rate of 1/6. None of the six antibiotics at a concentration of 50.00 mg/mL showed inhibitory effects on *A*. *baumannii* (Table 1 and S3 Fig). *A*. *camphorata* extracts exhibited remarkably potent inhibition against *A*. *baumannii*, maintaining its effectiveness even when the mass concentration was reduced to 12.50 mg/mL (Fig 1).

**Table 1:** Comparing the antimicrobial activity of *A*. *camphorata* extracts with the positive control.

Species	Chloramphenicol	Gentamycin	Ampicillin	Streptomycin	Tetracycline	Kanamycin	*A*. *camphorata* extract	Antibiotic Rate
*E. faecalis*	27.0±0.4	10.5±0.6	ND	18±0.7	12.5±0.7	15.0±0.8	10.3±0.5	1/6
*S. aureus*	24.5±0.3	12.0±0.5	ND	17.2±0.6	13.5±0.3	12.0±0.4	10.3±0.5	1/6
*P. aeruginosa*	ND	ND	ND	ND	8.5±0.6	ND	10.4±0.3	5/6
*A. baumannii*	ND	ND	ND	ND	ND	ND	13.5±0.4*	6/6
*K. pneumoniae*	28±0.2	13.0±0.3	ND	17.2±0.5	13.1±0.3	18.2±0.6	10.1±0.1	1/6
*E*. *coli*	ND	14.2±0.3	ND	16.5±0.2	14.5±0.4	11.8±0.8	13.9±0.4	2/6

ND: not detected; The mass concentration of each antibiotic and the extract of *A*. *camphorata*: 50.00 mg/mL; Values are the mean of n = 3 experiments, Inhibition zone diameter, measured in millimeters.

### Antibacterial activity of *A*. *camphorata* extracts against foodborne pathogens

**Fig 2:**
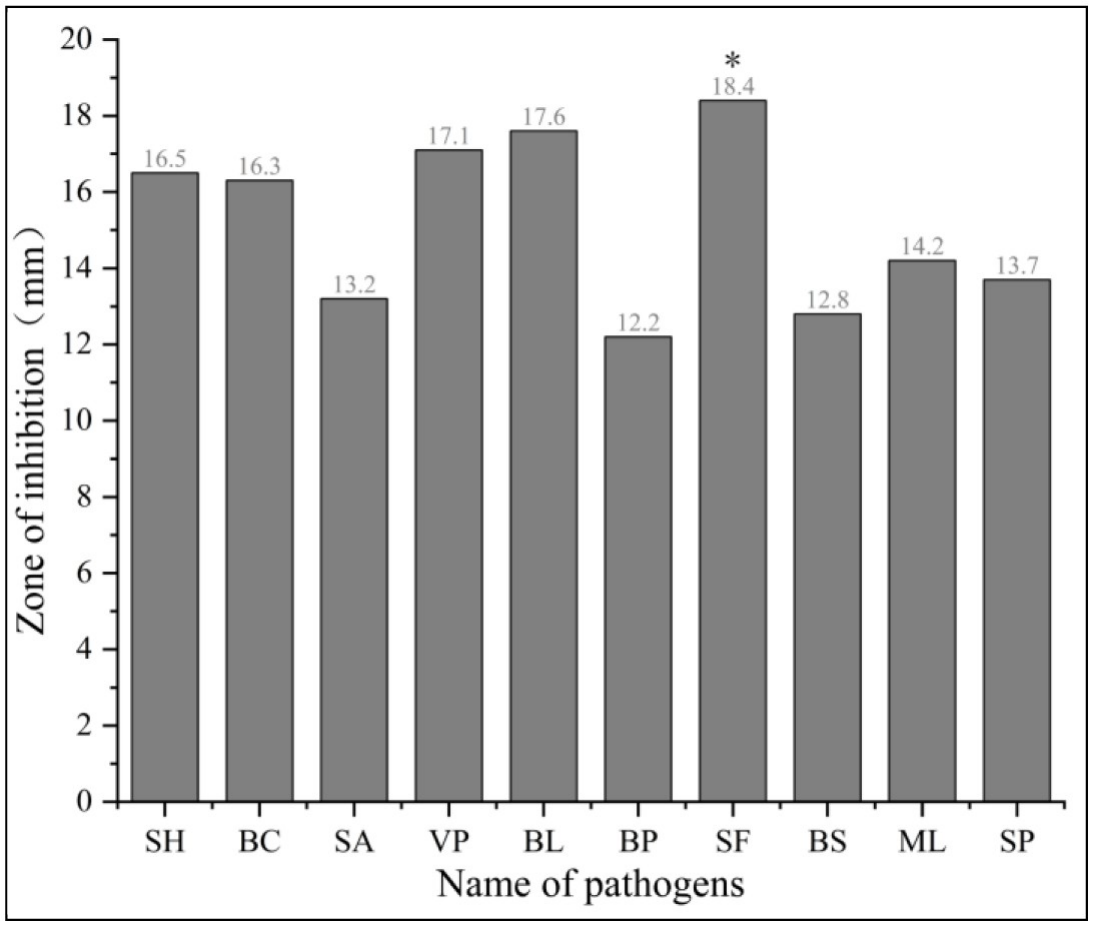
Antibacterial activity of *A*. *camphorata* extracts against foodborne pathogens.

Using the paper diffusion method, the antibacterial activity of the ethyl acetate extract of *A*. *camphorata* against 10 foodborne pathogens was evaluated. The extract showed significant inhibitory effect against all 10 tested bacteria at 50 mg/mL concentration, with the highest inhibitory effect observed against *Shigella flexneri*, which had an inhibition zone diameter of 18.3 mm. However, the extract’s effect on *Bacillus pumilus* was less potent, and an inhibition zone diameter of 12.3 mm was observed. The inhibitory activity of the remaining eight bacteria was better, with the inhibition zone ranging from 12.8 to 17.6 mm (Fig 2). These findings indicate that liquid fermentation of *A*. *camphorata* can generate a diverse array of active antibacterial substances with broad-spectrum activity.

### Isolation and identification of the antibacterial compound MBBD

The fermentation broth of *A*. *camphorata* was subjected to extraction by using ethyl acetate and concentrated using a rotary evaporator to yield 7.13 g of the extract from 4-L broth. The extract was fractionated through normal-phase silica gel column chromatography, each fraction was tested for antibacterial activity. When eluted with a mixture of petroleum ether and ethyl acetate in a 100:7 ratio, a white crystalline concentrate with antibacterial activity was obtained. The fraction containing the crystals was further purified through recrystallization. Preparative HPLC separation (95:5, methanol-water) led to the isolation of compounds AC-1 (12.22 mg, retention time = 1.526 min) and AC-2 (4.2 mg, retention time = 1.632 min). Antibacterial assays revealed that compound AC-1 had significant inhibitory activity against the tested bacteria, whereas compound AC-2 did not exhibit any activity (Table 2). Compound AC-1 was identified as MBBD by using NMR and MS methods, and its structural formula was determined to be 5-methyl-1,3-benzodioxole-4,7-diol (S4–S8 Figs).

**Table 2:** Separation and chemical analysis of *A*. *camphorata* extracts.

Compound	Retention time(min)	Peak area	Peak height	Peak width	Symmetry factor	Peak area ratio (%)
AC-1	1.526	2056.383	646.470	0.0510	1.041	89.724
AC-2	1.632	235.509	71.345	0.0505	0.896	10.276

### MIC of MBBD against pathogenic bacteria

**Fig 3:**
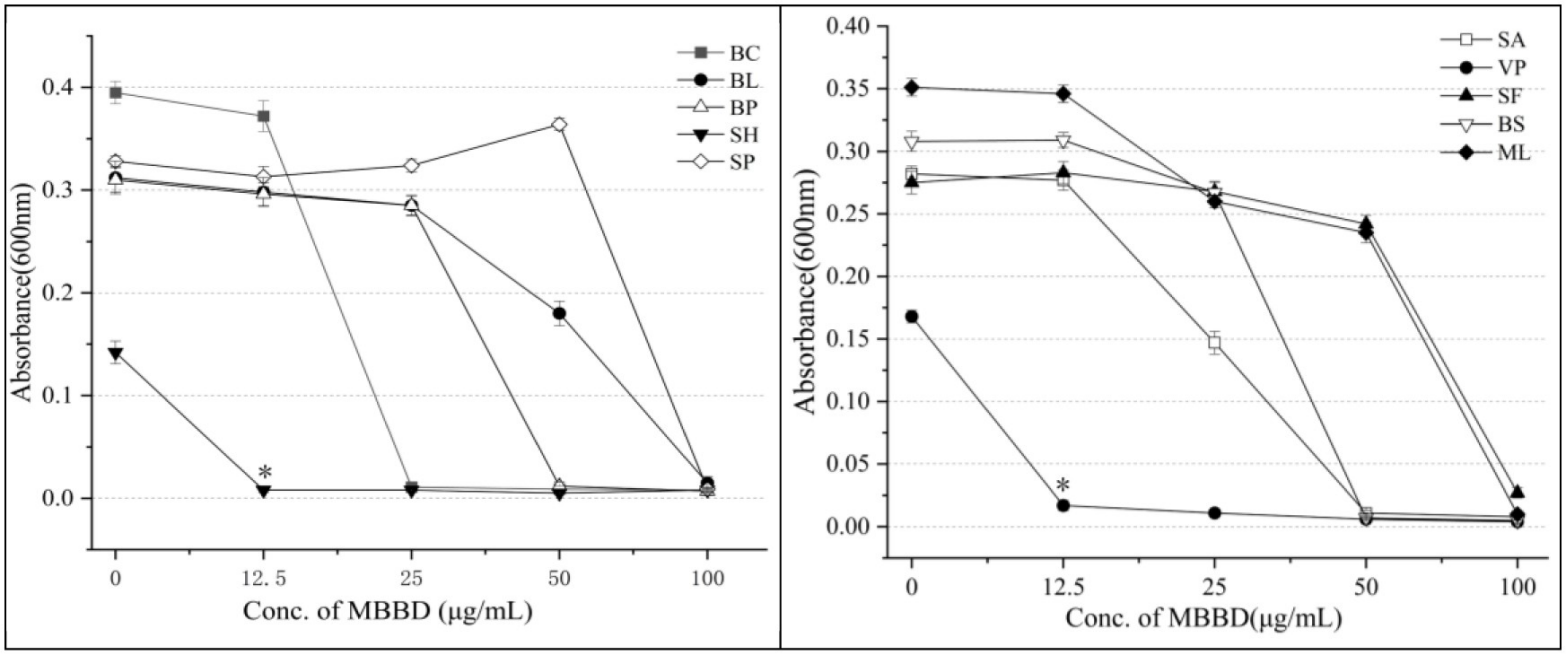
MIC of compound MBBD against foodborne pathogens. Values are the mean of n = 3 experiments**.**

**Fig 4:**
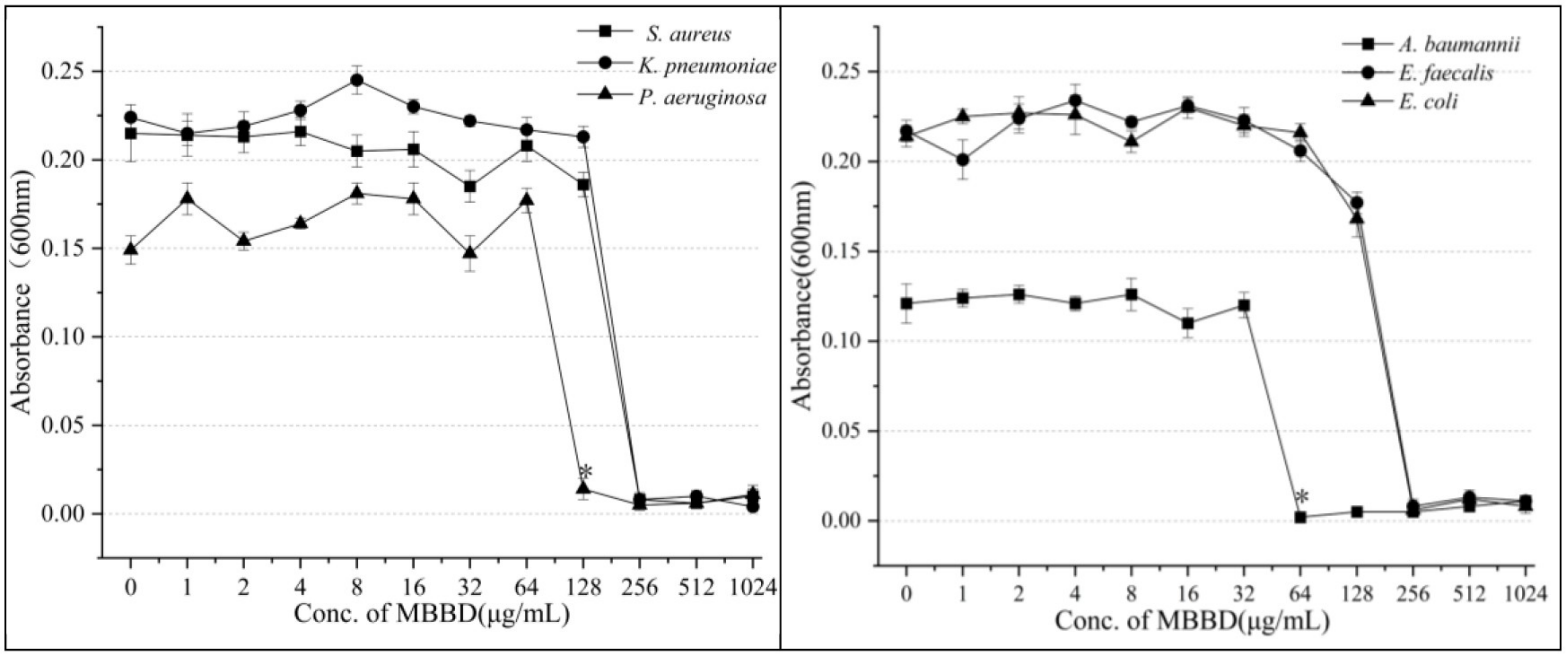
MIC of compound MBBD against resistant pathogenic bacteria. Data are reported as means ± SD of triplicate wells. Values are the mean of n = 3 experiments.

The compound MBBD has impressive antibacterial activity against various bacteria and well against both resistant pathogenic bacteria and foodborne pathogens. The microbroth dilution method is a standard technique for determining the MIC of an antibacterial compound against a pathogenic microbe. In this study, the MIC values of MBBD against foodborne pathogens ranged from 12.5 to 100 μg/mL. The lowest MIC value (12.5 μg/mL) was observed against *S*. *haemolyticus* and *V*. *parahaemolyticus*, followed by *B*. *cereus* (MIC = 25 μg/mL), *B*. *pumilus*, *B*. *subtilis*, and *Streptococcus agalactiae* (MIC = 50 μg/mL). However, the inhibition of *B*. *lentus*, *Salmonella paratyphi B*, *Shigella flexneri*, and *M*. *luteus* was relatively weak, with an MIC of 100 μg/mL (Fig 3). Moreover, the inhibitory effect of MBBD against drug-resistant pathogenic bacteria was also significant, with the lowest MIC value (64 μg/mL) observed against *A*. *baumannii*, followed by *P*. *aeruginosa* (MIC = 128 μg/mL). The MIC values of MBBD against *K*. *pneumoniae*, *S*. *aureus*, *E*. *faecalis*, and *E*. *coli* were 256 μg/mL (Fig 4). The consistent results obtained in triplicate are reassuring and suggest the robustness of the experiment.

### Cytotoxicity of MBBD

**Fig 5:**
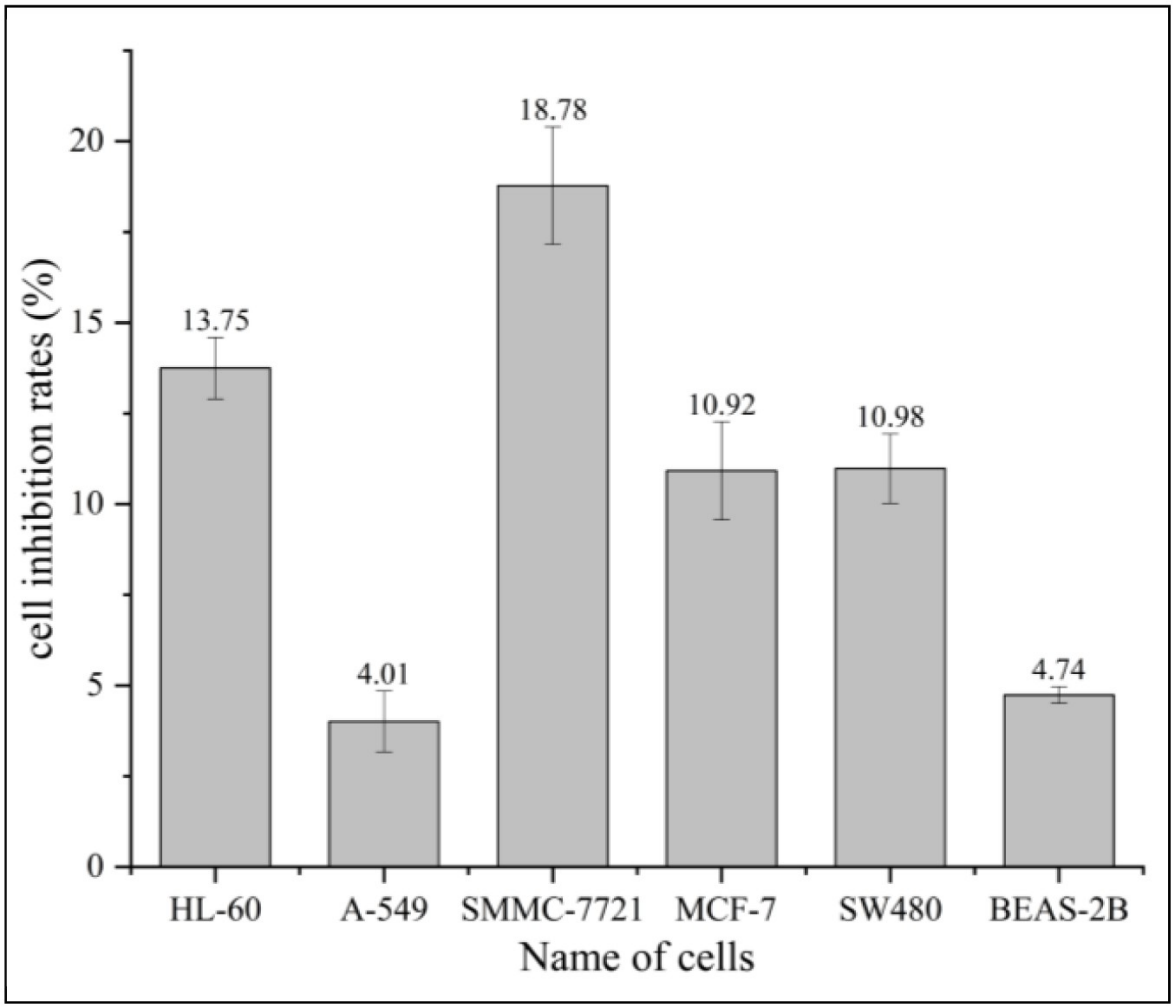
Cell inhibition rate of compound MBBD (40μM).

**Fig 6:**
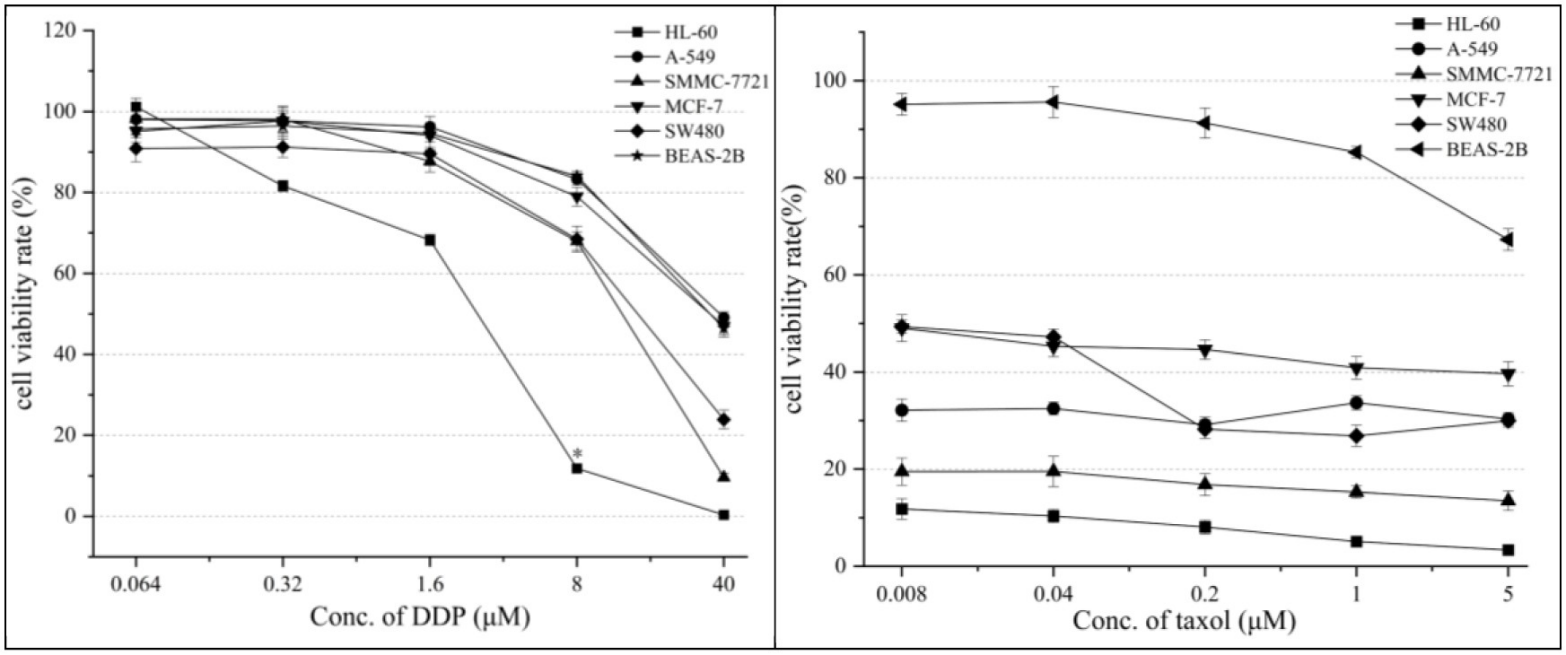
Cell inhibition rate of positive control group DDP and taxol.

MBBD was insensitive to five types of human cancer cells and exhibited no cytotoxicity against human normal lung epithelial cells BEAS-2B (S9 Fig). The MTS assay showed that the cell inhibition rates of MBBD (40 μM) against HL-60, A-S49, SMMC-7721, MCF-7, SW480, and BEAS-2B were all <20% (Fig 5). This suggests that MBBD can potentially serve as a therapeutic agent that selectively targets pathogenic bacteria without affecting healthy human cells. Furthermore, the positive controls cisplatin DDP and taxol significantly inhibited the tested human cancer cells (Fig 6).

## Discussion

*A*. *baumannii* is a pathogenic bacterium that commonly causes infections in clinical settings, and its resistance to antibiotics has been increasing due to acquired resistance and antibiotic misuse. Owing to the emergence of multidrug-resistant *A*. *baumannii* (MDRAB), extensively drug-resistant *A*. *baumannii*, and even pan-drug-resistant *A*. *baumannii*, the number of antibiotics available for clinical treatment is limited, which has thus created an urgent need for novel antibiotics [24]. Polymyxin B is currently the drug of choice for treating MDRAB, but its use is limited because of nephrotoxicity, and drug resistance has been reported recently [25]. Cefiderocol, a new iron-carrying cephalosporin, is in phase III clinical trials and has shown robust activity against MDRAB. However, cefiderocol, when used alone, may result in the development of drug-resistant bacteria and may have some side effects, such as diarrhea, rash, blood in urine, a positive urine test for red blood cells, and an increased plasma white blood cell count [26–28].

Research into the development of new antibacterial drugs from fungi is gaining momentum because of the abundant resources available, lower adverse effects, and lower likelihood of drug resistance. Studies have revealed that altenusin, a compound isolated from the endophytic fungus *Alternaria tenuissima* PC-005 of *Polygonum cephalicum*, exhibited good antibacterial activity against *A*. *baumannii* with a MIC value of 125 μg/mL [29]. Similarly, researchers isolated an active compound, Flindersine (2,6-dihydro-2,2-dimethyl-5H-pyrano [3,2-c] quinoline-5-one-9cl), from the ethyl acetate extract of *Toddalia asiatica*. This compound exhibited MIC values of 125 μg/ml against *A*. *baumannii* [30]. Additionally, a newly discovered naturally-occurring hydroquinone, α,2,5-trihydroxyacetophenone, was extracted from the marine-derived fungus *Pseudopithomyces maydicus* PSU-AMF350. This compound demonstrated antibacterial efficacy against *A*. *baumannii*, with an MIC value of 200 μg/mL [31]. In this study, MBBD, isolated and purified from the *A*. *camphorata* fermentation broth, exhibited a significant inhibitory effect on *A*. *baumannii* with a MIC value of 64 μg/mL. Additionally, it exhibited a stronger antibacterial effect than altenusin, Flindersine and α2,5-trihydroxyacetophenone. Notably, the potential use of MBBD as an antibiotic for *A*. *baumannii* treatment is promising and could offer a new approach to combat this challenging pathogen.

**Fig 7:**
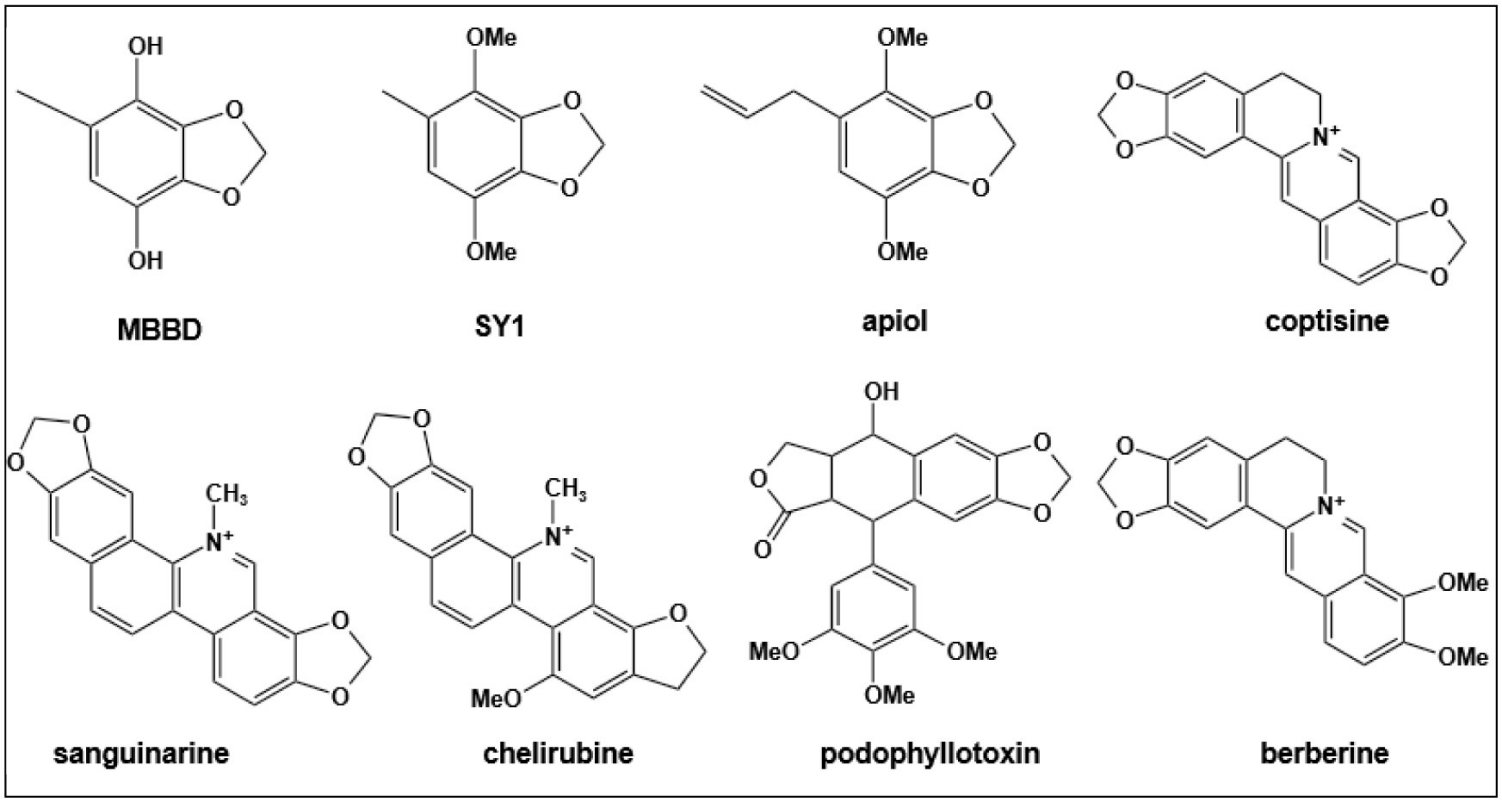
Chemical structure of MBBD and its analogs.

MBBD belongs to the benzodioxole class and was first isolated from *A*. *camphorata* fermentation broth by Taiwanese researchers in 2011. It exhibited good antioxidant activity [32]. In the same year, researchers successfully isolated and purified another benzodioxole-like compound known as SY1 (4,7-dimethoxy-5-methyl-1,3-benzodioxole) from *A*. *camphorata* substrates. SY1 exhibited anticancer activity [33]. Benzodioxole structures are less common in fungi, but they are more common in plants. For example, apiol, sanguinarine (SAG), chelirubine, coptisine, berberine, and podophyllotoxin (Fig 7) are known to contain benzodioxole analogs with antibacterial properties. Berberine, when combined with other antibiotics, effectively killed MRSA by inhibiting phenol-soluble regulatory protein aggregation into amyloid fibrils, thereby disrupting MRSA biofilms [34]. SAG, which is found in the *Papaveraceae*, *Berberidaceae*, and *Ranunculaceae* families, possesses antioxidant, anti-inflammatory, and antiproliferative properties against various malignancies. In 2020, a study reported a significant inhibitory effect of SAG against *Providencia rettgeri*, with a MIC value of 7.8 μg/mL [35]. Chelerythrine (CHE) is a natural benzophenanthridine alkaloid derived from traditional medicinal plants of the *Papaveraceae* family and *T*. *asiatica* [36]. In prior research, CHE isolated from *T*. *asiatica* roots exhibited antibacterial activity against β-lactamase-resistant *Staphylococcus* by disrupting the bacterial cell wall, cell membrane, and inhibiting protein biosynthesis [37]. Subsequent investigations conducted by other scientists demonstrated that CHE also hindered the growth of carbapenem-resistant *Serratia marcescens* (MIC = 125 μg/mL /mL), leading to disruptions in cell membrane integrity and significant alterations in cell morphology [38]. In our study, MBBD displayed significant inhibitory action against both ESKAPE pathogens (MIC = 64–256 μg/mL) and foodborne pathogens (MIC = 12.5–100 μg/mL). These studies indicate that benzodioxole analogs have a wide range of biological activity. In summary, MBBD containing the benzodioxole structure can be considered an effective approach for the prevention or inhibition of *A*. *baumannii*. Furthermore, it exhibits no toxicity toward human normal lung epithelial cells, potentially making it a comparatively safer option. However, achieving large-scale production of MBBD poses several significant challenges that require careful consideration. First and foremost, identifying a culture medium capable of promptly and efficiently inducing MBBD production in *A*. *camphorata* is of paramount importance. Additionally, gaining a comprehensive understanding of the entire biosynthetic pathway, including all relevant enzymes and genes, necessitates the application of ’omics’ methodologies, which encompass genomics, transcriptomics, proteomics, and metabolomics. This comprehensive elucidation is poised to facilitate precise regulatory and manipulative strategies for optimizing the biosynthetic process, ultimately resulting in enhanced production efficiency [39]. Significantly, prior investigations have unveiled distinct benzodioxole derivatives demonstrating noteworthy anticancer and antibacterial properties, hinting at a conceivable correlation between these effects and the compounds’ DNA-binding capabilities [40]. As our research progresses, we are dedicated to embarking on a comprehensive exploration of the potential mechanisms underlying MBBD’s antibacterial activity.

Nowadays, the fruiting bodies of *A*. *camphorata* are in great demand and very expensive due to host specificity, rarity in nature, and the difficulty of artificial cultivation. Malic acid and succinic acid derivatives are the primary bioactive compounds in the liquid fermentation of *A*. *camphorata*. Among these compounds, antrodin A exhibits excellent inhibitory activity against the protease of the hepatitis C virus, while antrodin B and C demonstrate significant cytotoxicity against lung cancer cell lines [41]. Liquid fermentation allows for better control of the cultivation environment. Some researchers have found that supplementing the culture medium with p-hydroxybenzoic acid, eugenol, and coenzyme Q0 during the liquid fermentation of *A*. *camphorata* can promote the production of antroquinonol, a compound with remarkable anticancer properties [42]. Additionally, the addition of petroleum ether extracts from camphor trees to the culture medium has been shown to significantly increase the total triterpene yield of *A*. *camphorata*. Therefore, deep fermentation technology stands out due to its advantages, including short cycles, high efficiency, low cost, and ease of scalable production [43–45]. In this study, *A*. *camphorata* was cultured using liquid fermentation because it is less time-consuming and cost-effective. MBBD (122.8 mg/4 L) with broad-spectrum antibacterial activity was isolated and purified from the *A*. *camphorata* fermentation broth. Therefore, liquid fermentation of *A*. *camphorata* has the potential to yield numerous antimicrobial compounds. In conclusion, MBBD can serve as a reference for developing drugs to combat *A*. *baumannii* and can play a crucial role in the application of liquid fermentation of *A*. *camphorata* and the development of new antibacterial drugs.

## Conclusion

In this study, compound MBBD was isolated and identified from the fermentation broth of *A*. *camphorata*. MBBD exhibited inhibitory activity against drug-resistant bacterial strains, including *A*. *baumannii*, *P*. *aeruginosa*, *K*. *pneumoniae*, *S*. *aureus*, *E*. *faecalis*, *E*. *coli*, as well as 10 foodborne pathogens. In particular, it demonstrated a significant inhibitory action against highly resistant *A*. *baumannii*. Additionally, MBBD was non-toxic to the normal human lung epithelial cells BEAS-2B. MBBD exhibits broad-spectrum antibacterial activity, particularly demonstrating excellent inhibitory effects against *A*. *baumannii*. MBBD may be good candidates for new antimicrobial drugs.

## Supporting information

S1 Fig *Antrodia camphorata* (culture). (DOCX)

S2 Fig Culture filtrate of *A*. *camphorate*. (DOCX)

S3 Fig Antibacterial activity *Antrodia camphorata*. Gen. represents Gentamicin, Amp. represents Ampicillin, Str. represents Streptomycin, Tet. represents Tetracycline, Kan. represents Kanamycin, BD represents *A*. *camphorata* extracts (50.00 mg/mL), BD-1 represents *A*. *camphorata* extracts (25.00 mg/mL), BD-2 represents *A*. *camphorata* extracts (12.50 mg/mL). The figures, from left to right and top to bottom, represent *A*. *baumannii*, *S*. *aureus*, *P*. *aeruginosa*, *E*. *coli*, *K*. *pneumoniae*, and *E*. *faecalis*.
(DOCX)

S4 Fig Compounds separation through chromatography. (DOCX)

S5 Fig HPLC of MBBD. (DOCX)

S6 Fig C spectrum of MBBD. (DOCX)

S7 Fig H spectrum of MBBD. (DOCX)

S8 Fig MS of MBBD. (DOCX)

S9 Fig Cytotoxicity photos. MBBD was not cytotoxic to human normal lung epithelial cells BEAS-2B.
(DOCX)
