# Supplementary figures and images for: Isolation and identification of bioactive compounds from Antrodia camphorata against ESKAPE pathogens
Source: PLoS One. 2023 Oct 27;18(10):e0293361. doi: 10.1371/journal.pone.0293361 (PMC10610075; doi:10.1371/journal.pone.0293361)

**S1 Fig.**

| **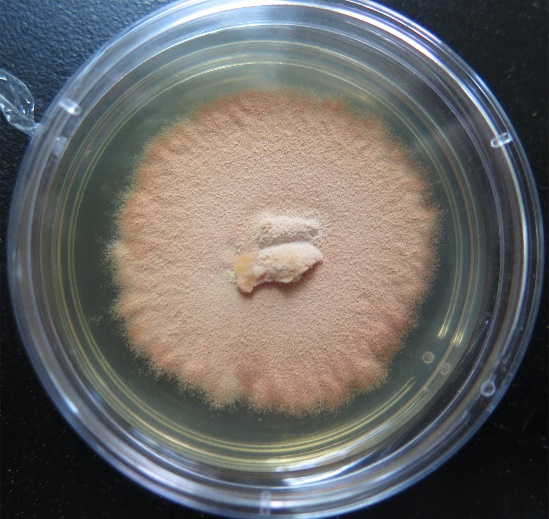** | **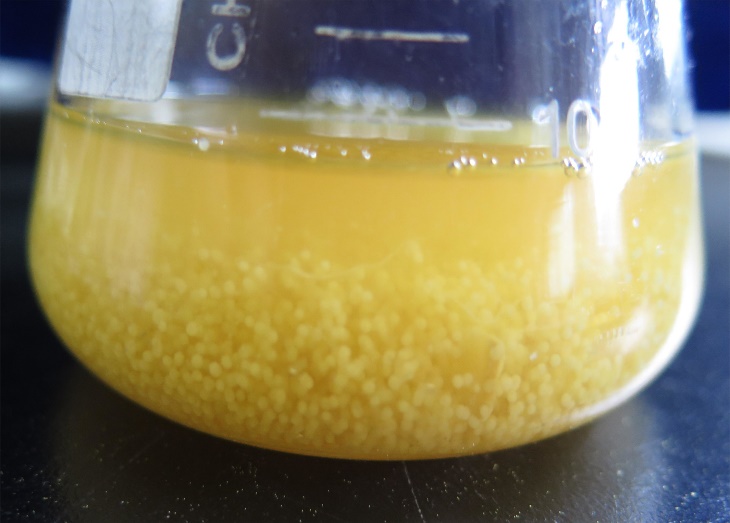** |
| --- | --- |

Supplement: S1 Fig — (DOCX) [file pone.0293361.s001.docx]

**S2 Fig.**

| **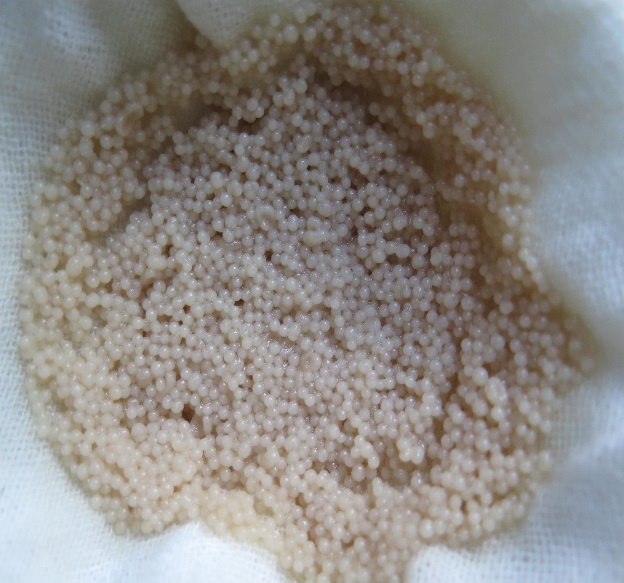** | **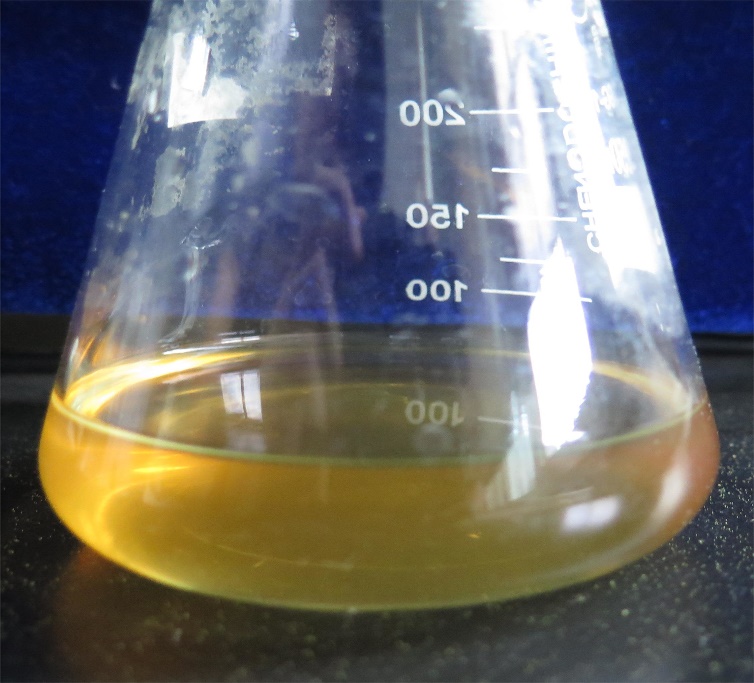** |
| --- | --- |

Supplement: S2 Fig — (DOCX) [file pone.0293361.s002.docx]

**S3 Fig.**

| **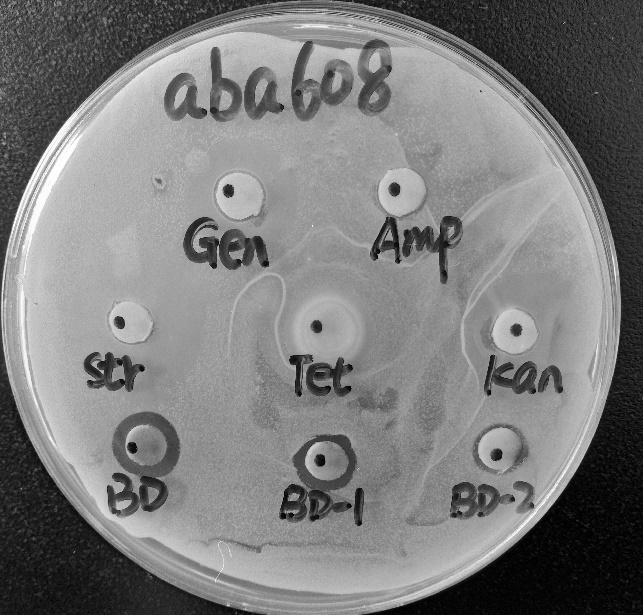** | **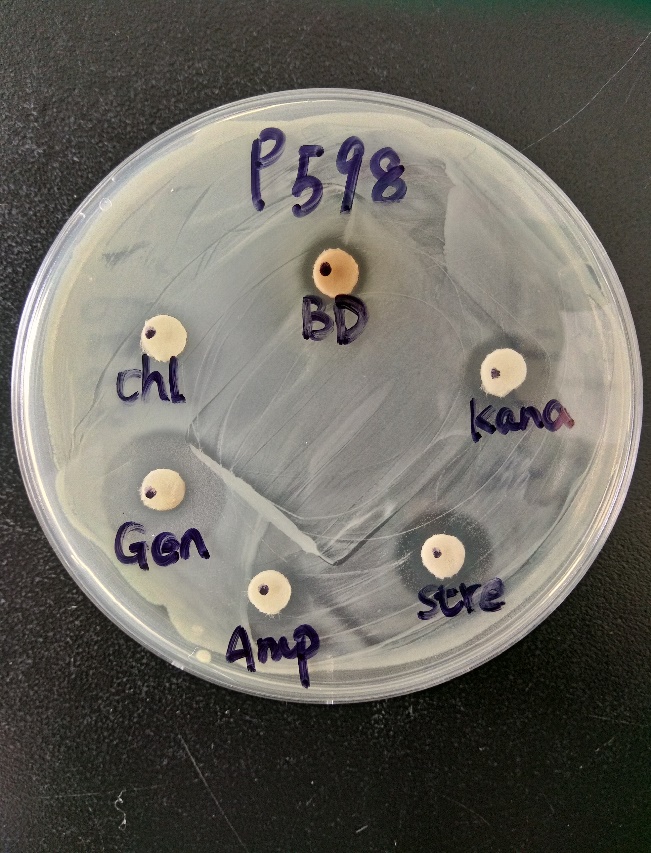** |
| --- | --- |
| 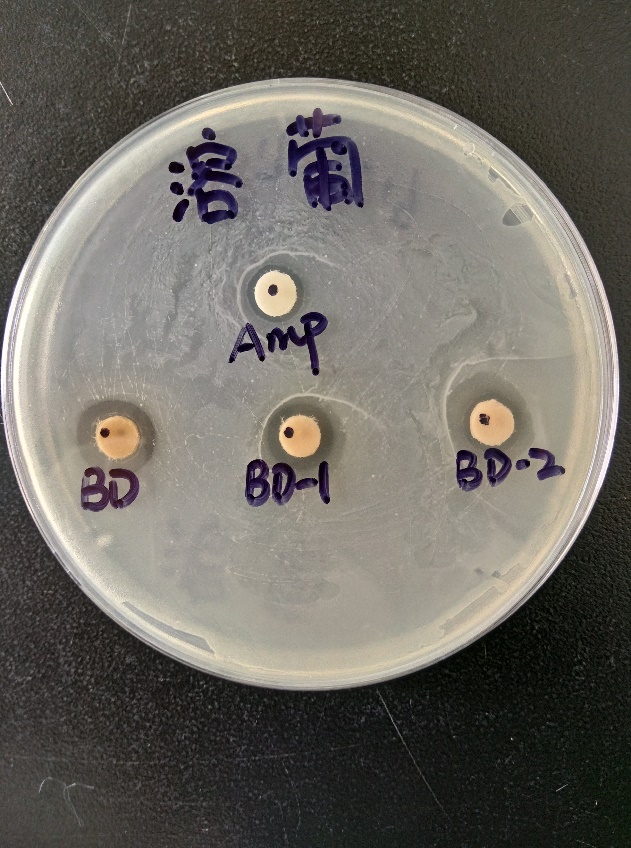 | **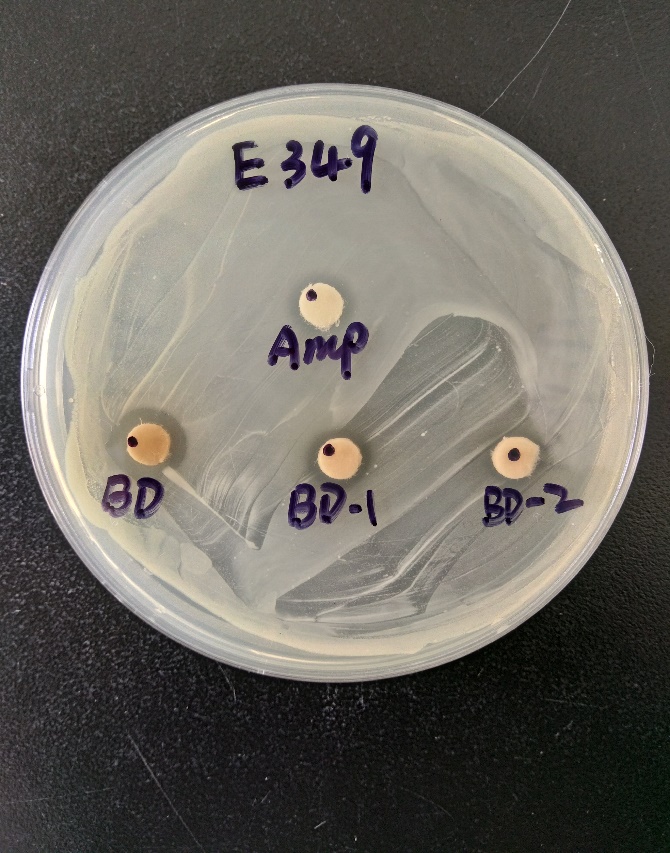** |
| **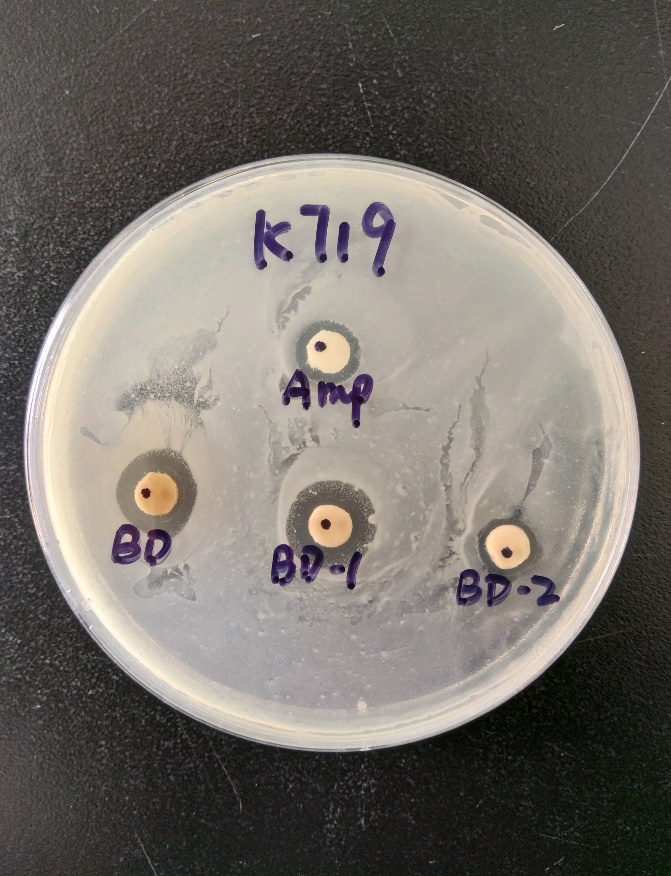** | **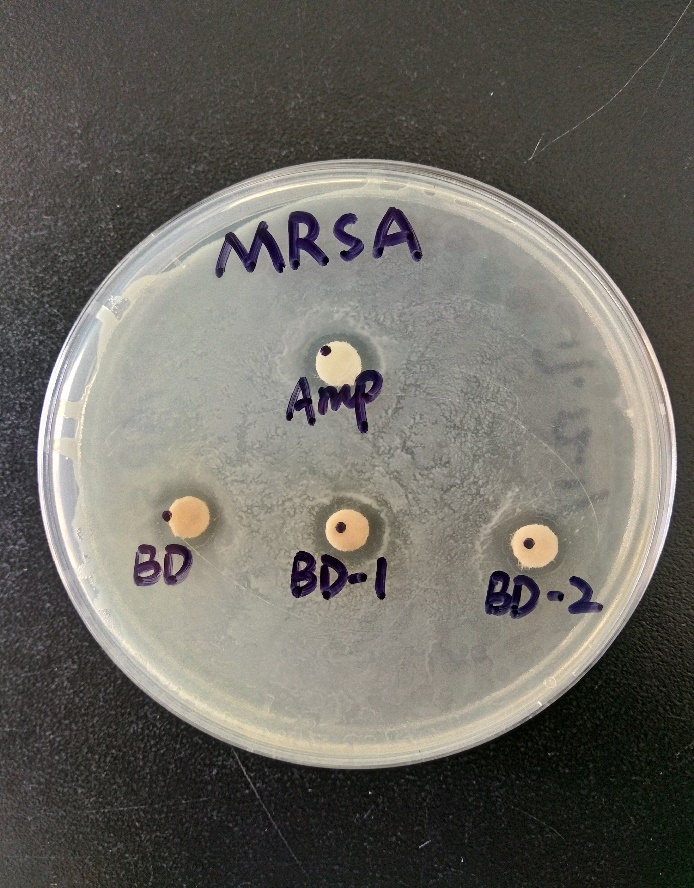** |

Supplement: S3 Fig — Gen. represents Gentamicin, Amp. represents Ampicillin, Str. represents Streptomycin, Tet. represents Tetracycline, Kan. represents Kanamycin, BD represents A. camphorata extracts (50.00 mg/mL), BD-1 represents A. camphorata extracts (25.00 mg/mL), BD-2 represents A. camphorata extracts (12.50 mg/mL). The figures, from left to right and top to bottom, represent A. baumannii, S. aureus, P. aeruginosa, E. coli, K. pneumoniae, and E. faecalis. (DOCX) [file pone.0293361.s003.docx]

**S4 Fig.**

| **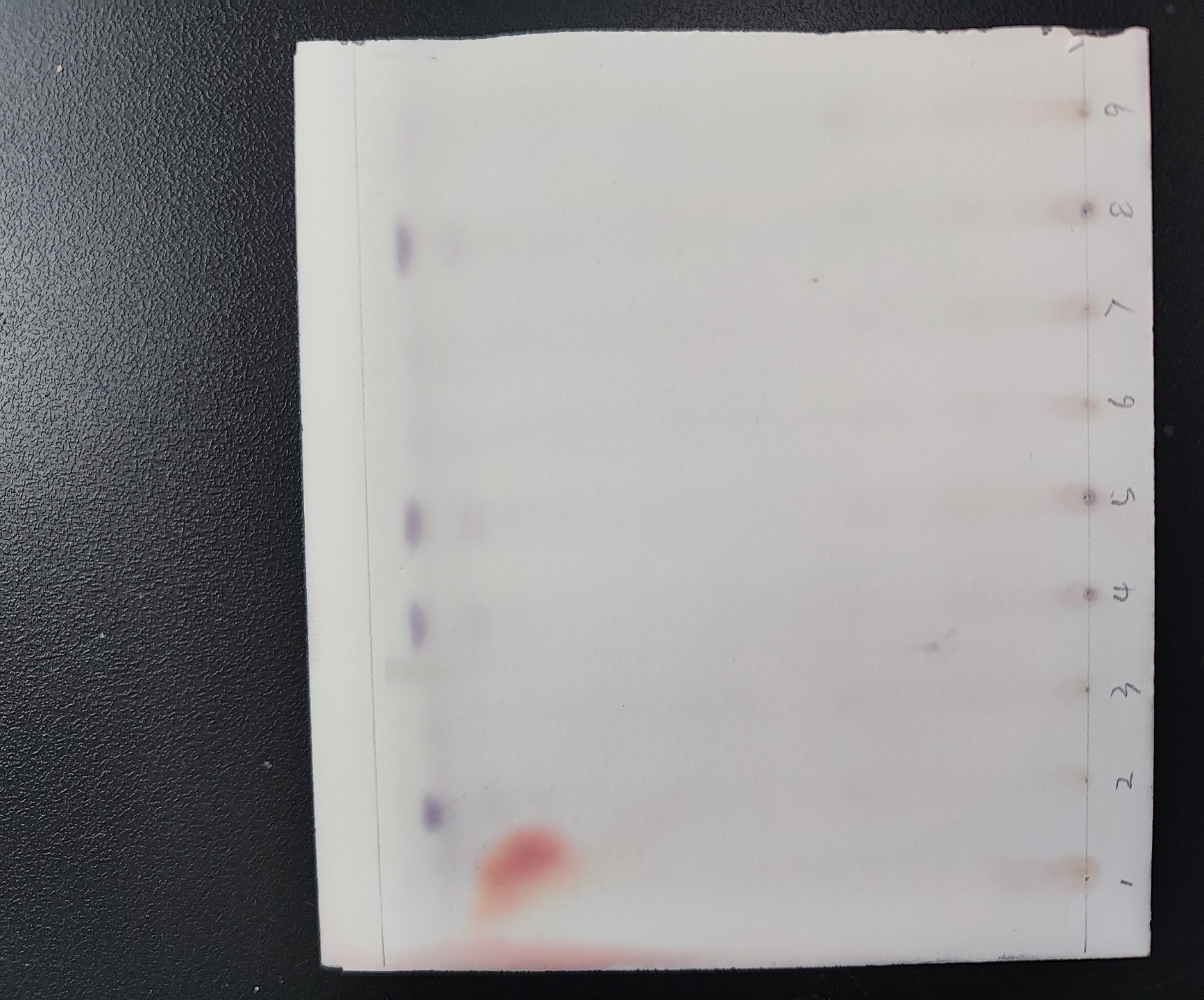** |
| --- |

Supplement: S4 Fig — (DOCX) [file pone.0293361.s004.docx]

**S5 Fig.**

**
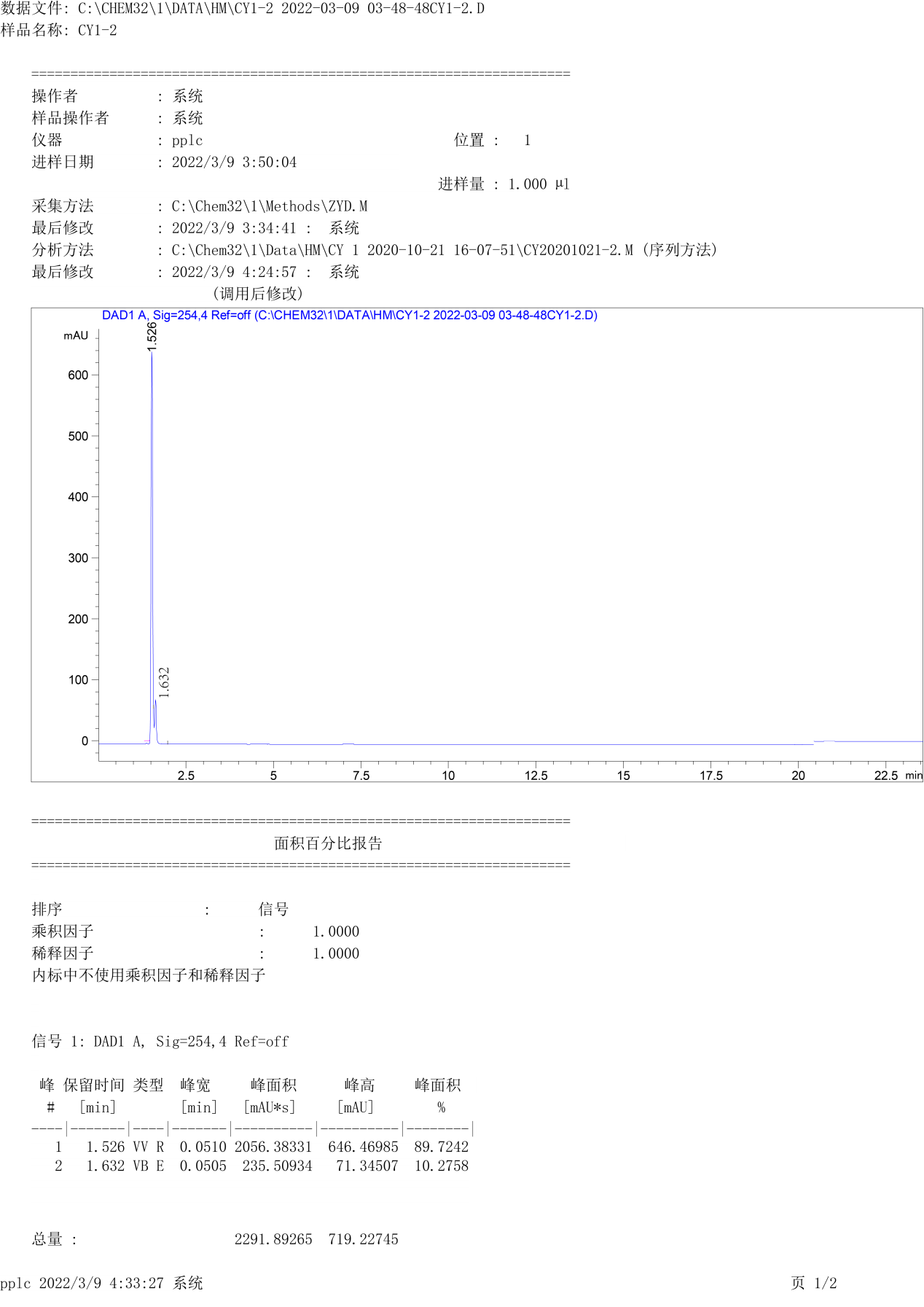
**

Supplement: S5 Fig — (DOCX) [file pone.0293361.s005.docx]

**S6 Fig.**


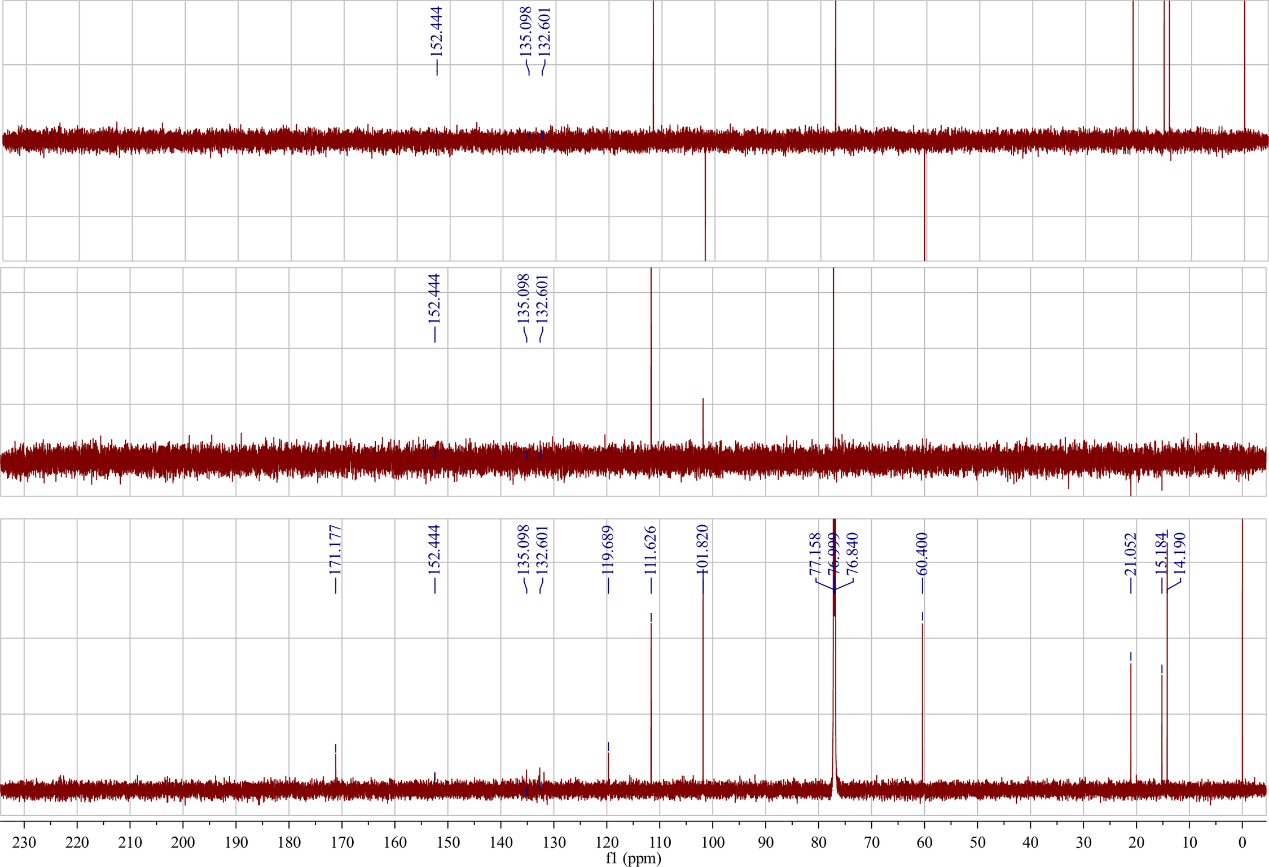

Supplement: S6 Fig — (DOCX) [file pone.0293361.s006.docx]

**S7 Fig.**


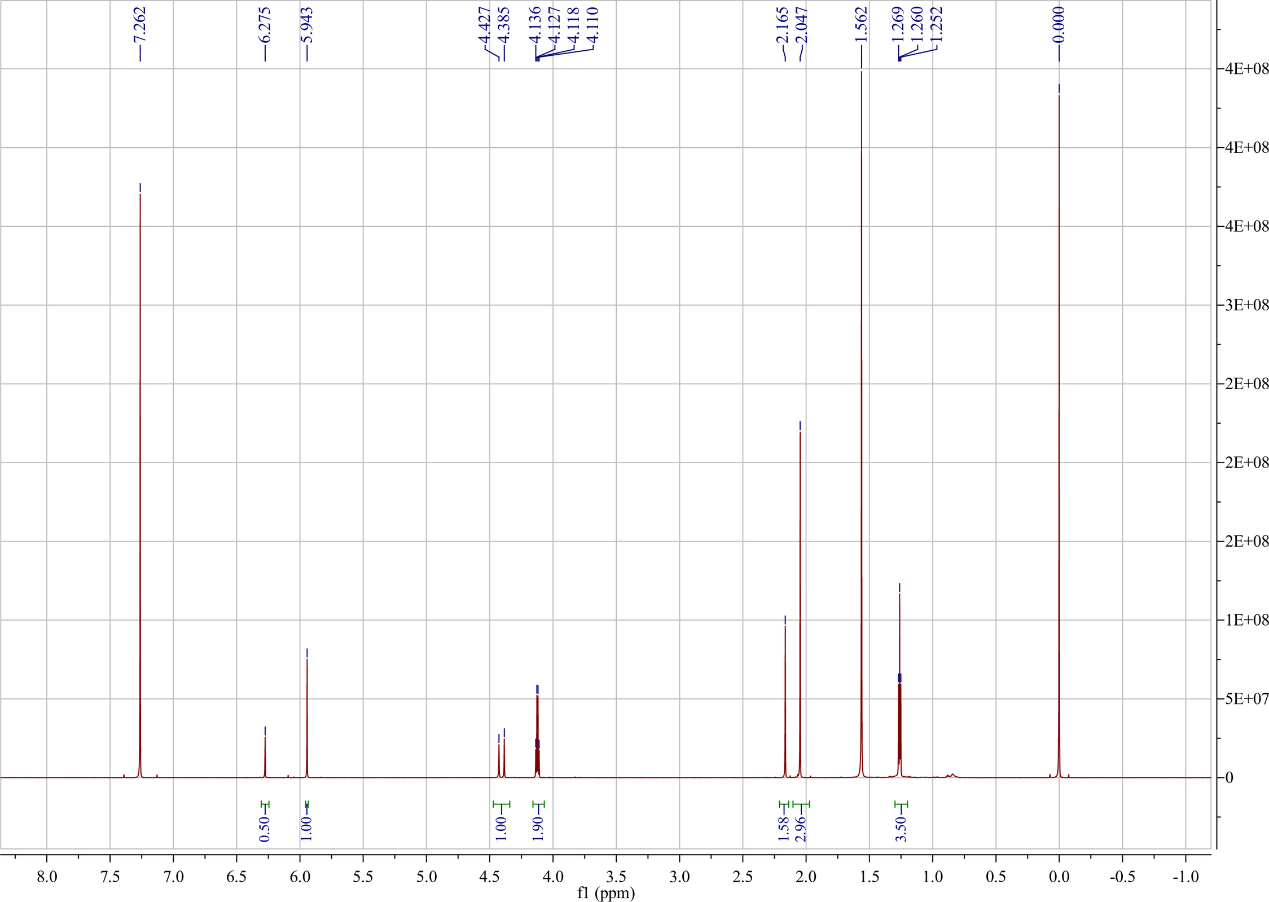

Supplement: S7 Fig — (DOCX) [file pone.0293361.s007.docx]

**S9 Fig.**


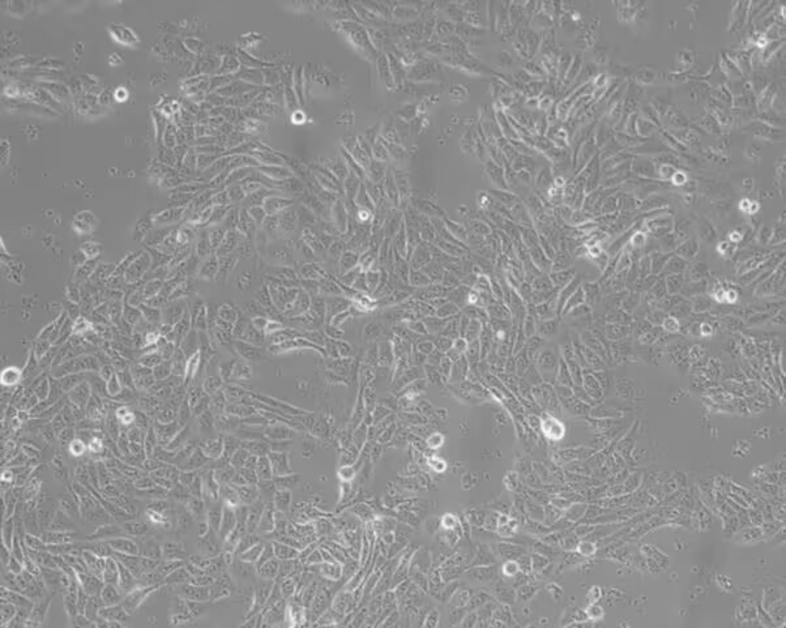

Supplement: S9 Fig — MBBD was not cytotoxic to human normal lung epithelial cells BEAS-2B. (DOCX) [file pone.0293361.s009.docx]
